# Evaluating the Relationship between Competition and Productivity within a Native Grassland

**DOI:** 10.1371/journal.pone.0043703

**Published:** 2012-08-21

**Authors:** Jonathan A. Bennett, James F. Cahill

**Affiliations:** Department of Biological Sciences, University of Alberta, Edmonton, Alberta, Canada; Lakehead University, Canada

## Abstract

Ideas about how plant competition varies with productivity are rooted in classic theories that predict either increasing (Grime) or invariant (Tilman) competition with increasing productivity. Both predictions have received experimental support, although a decade-old meta-analysis supports neither. Attempts to reconcile the conflicting predictions and evidence include: expanding the theory to include other conditions (e.g. stress gradient hypothesis), development of indices to differentiate either the ‘intensity’ or ‘importance’ of competition, a focus on resource supply and demand, and explicit recognition that both growth and survival may exhibit different relationships with productivity. To determine which of these theories accurately predict how competition varies with productivity within a native grassland site, we estimated competitive intensity and relative competitive importance using 22 species across the range of productivity naturally occurring within that site. Plant performance was measured as survival and size with and without neighbours and the local environment was quantified according to variability in standing crop, gross water supply, and net water supply. On average, neighbours weakly facilitated seedling survival, but strongly reduced seedling growth. For both seedling survival and growth, relative competitive importance and competitive intensity declined with some measure of productivity; neighbour effects on survival declined with standing crop, while effects on growth declined with gross water supply. These results add to the growing evidence that plant-plant interactions vary among life history components with different life history components contingent upon separate environmental factors. Although the range of productivity measured in this study was not large, our results do not support the theories of Grime or Tilman. However, our results are consistent with the meta-analysis and parts of other theories, although no single theory is capable of explaining the entirety of these results. This suggests that, at least in moderately productive grasslands, new theory needs to be developed.

## Introduction

Plant competition is regularly seen as a factor influencing the structure of natural communities. However, the intensity of competition is not constant through space nor time [1], [2], [3], [4], [5], and understanding the factors that influence its intensity is critical to understanding the assembly of plant communities. One factor that plant ecologists have long focused on is the relationship between plant competition and productivity. This issue has been studied for so long and so intensely that it is a common topic in introductory ecology textbooks [6], [7], [8], [9].

Textbook authors often present competition-productivity relationships in the context of the theories of Grime [10], [11], [12], Newman [13], and Tilman [3], [14]. Many aspects of the disagreements amongst these authors are presented elsewhere [15], [16], [17]. In brief, Grime [10], [11], [12] asserted that competition and competitive exclusion is only important in structuring communities at high productivity, with “stress” more important at low productivity. Newman [13] suggested that competition was strong at both low and high productivity, though it switched from root to shoot competition as resources increased. However, Newman (1973) agreed that it was likely that only shoot competition would reduce species diversity, a prediction consistent with later experimental work [18], [19]. Tilman [3], [14] also suggested the intensity of competition would be invariant along productivity gradients, but expected competition to be an important structuring force for communities at all levels of productivity [3]. Thus these sets of theories make separate predictions about when competition will be strong, and when it will be important for structuring communities along productivity gradients (Table 1).

**Table 1:** C–P relationship predicted for each major theory and the Goldberg meta-analysis.

Response metric	Gradient	Grime	SGH	Newman	Tilman	Goldberg	Davis	This study
Intensity	Standing crop/Gross supply	+	+	0	0	−	0/−	−
	Net supply						−	0
Competitive frequency	Standing crop/Gross supply		+					−/0
Relative importance	Standing crop/Gross supply	+	+			−		−
Demographic importance	Standing crop/Gross supply	+	+	+	0	−		

**Note:** Predictions are based on Grime [10], [11], [12], Bertness and Callaway [37], Maestre *et al.* [40], Newman [13], Tilman [3], [14], Goldberg *et al.* [1], and Davis et al. [5]. Cells containing a + mean that we expect increasing competition along the gradient, cells with a 0 mean that we expect a non-significant relationship, and cells with a − mean we expect a negative relationship. If a cell is left blank, then that particular metric or gradient does not apply to that theory. The column labelled this study refers to our findings and will be explained further in the results and discussion.

Debate and data focused on competition-productivity (C–P) relationships has appeared in the scientific literature for over twenty years [1], [16], [17], [20], [21], [22], [23], [24], [25], [26], [27], [28], [29], [30], [31], including experimental efforts which have spanned continents [32]. One of the first meta-analyses in ecology was published by Goldberg and colleagues [1] focused on this issue. They found the intensity of competition for plant survival and growth *declined* with productivity; an outcome that supports none of the theories described above. Nonetheless, these unexpected and synthetic empirical results do not appear with the same frequency as the original theories in modern textbooks, even though another meta-analysis found similar results [33]. Despite the relative commonness of competition declining with productivity, these results are often disputed [34], [35] and Grime and Tilman typically remain the starting point for new theory on this topic [16], [36].

One such expansion of Grime’s theory, the stress-gradient hypothesis (SGH), relates facilitation to stress [37], [38], [39], where the level of stress within a community is often approximated by measuring productivity [15], [40] and facilitation is measured using metrics similar to those used to study competition. Thus, in practice, SGH studies are empirically identical to C–P studies, although the research focus is more often on the outcome of pair-wise interactions rather than effects on community structure [40]. SGH is often only applied to low productivity areas, with the primary prediction being that facilitation should be most common [37], [40], intense [39] or important [40], [41] at high to intermediate stress and thus that competition should be lowest in unproductive areas. Attempts have been made to apply this hypothesis to more productive areas [42], including an additional, yet little explored, aspect of the initial SGH which predicts that associational defences can cause facilitation to increase with productivity when herbivore pressure is intense [37]. Thus the predictions of SGH are multi-faceted and can be consistent with the predictions of Grime, Newman, Tilman, or the results presented by Goldberg, depending on the range of productivity encountered.

Alternate hypotheses regarding the relationship between competition and productivity focus not on the biomass of neighbours, but on the supply and demand of resources [5], [43]. In these theories, it is either difference between supply and demand [5] or the ratio of supply and demand [43] that determines the outcome of competitive interactions, with competition declining as either net resource supply (supply - demand) or the ratio of supply to demand increases. Thus in both these cases, competition can decrease with productivity. Although these theories are rooted in the foundations of plant ecology [44], they do not receive as much attention as theories relating to the work of Grime and Tilman.

C–P relationships can be expected to vary as a function of many factors. For example, plant survival and growth may respond differentially to the presence of neighbours [40], [45], [46], [47], and may have differential effects on species exclusion [45]. These differences are rarely addressed by theory [40], [48], though they have been addressed empirically [1], [5], [49]. Additionally, theory predicts different relationships for competitive *intensity* and *importance* [15], [16], [17], [20], [30], [40]. Intensity is typically defined as the absolute magnitude of an effect [17], [50], while importance has highly variable and contentious points of view among researchers. For this manuscript, we will measure importance as the magnitude of competition relative to maximum plant performance [15], [17], [51]. We recognize competitive importance can also be viewed in terms of the ultimate effects of competition on population growth rates [52], competitive exclusion and community structure [53], [54]. When differentiating between these definitions of importance, we will refer to them as relative and demographic importance respectively. However, demographic importance is difficult to measure in a perennial plant community given the long life spans of some plants and as such we cannot test them with the data at hand [but see 53 for a potential method of looking at community consequences of competition].

Finally, though C–P relationships are presented as an aspect of a community, they may be highly variable among species within a community. To date, most experimental studies of competition have used one or a small number of *phytometers*, species intended to be representative of the entire community [5], [15], [23], [26], [55], with exceptions including studies such as Wilson and Tilman [29] with eight and Callaway *et al.* [56] with more than sixty. What is lacking are experimental studies with a large number of species measuring multiple responses within a single community - allowing a true test of the overall relationship within that community.

The aim of this paper is to investigate the predictions of each of the aforementioned theories as outlined in Table 1 for the plant community within a single site using a large number of species. We attempt to address some of the potential causes of disagreement among empirical results by differentiating between size and survival using indices of both competitive intensity and competitive importance among plots naturally varying in both neighbour biomass and resource availability.

## Methods

### Study Site and Species

The study occurred in an unbroken and unseeded 50 ha section of native prairie at the University of Alberta research ranch at Kinsella, Alberta, Canada (53°05′N, 111°33′W). The field site is a savannah type habitat with mixed grass prairie interspersed with stands of aspen (*Populus tremuloides)*. Standing crop within the prairie area naturally varies from 100 to 800 g/m^2^, with *Hesperostipa curtiseta* and *Festuca hallii* being the most common species at low to moderate productivity, switching to *Poa pratensis* and *Galium boreale* at moderate to high productivity (J.A. Bennett, unpublished data). The site is co-limited by water and nitrogen [57], although competitive intensity is more closely linked to water availability [53]. Competition is generally intense; often reducing plant growth by approximately 90% during seedling establishment [57], [58] and 50% in established plants [53], [59]. Neighbour effects on seedling survival are typically near neutral [58], [59], except during extended drought, when competition greatly increases seedling mortality [60].

The site receives an average of 418 mm total precipitation annually, which includes 155 mm of snow and rain from first snowfall through spring and 217 mm rain over the summer months (June through August). However, there is never an average year in a continental grassland, and precipitation was approximately 75% of average both leading up to and during the experiment. Daily temperatures over the experimental period were similar to the thirty year average, with mean daily temperatures of 15.2°C, average highs of 22.3°C and lows of 8.0°C. Though the site has historically been grazed by cattle, grazing had been halted four years prior to the onset of the experiment and did not occur for the duration of the study. Insect herbivory has little effect on the plant community at this site [61], while grazing by free-ranging ungulates (e.g. deer) and small mammals is infrequent (J.A. Bennett, personal observation).

We recognize that the range of productivity within our study is smaller than would be found in a transcontinental study [32], [56]. However, none of the C–P theories except the SGH [39], [40] suggest they operate only under specific ranges of productivity, and even the SGH has been extended into more productive environments [37], [42]. We also recognize that non-linearities in relationships [51], [55] would be difficult to detect using narrow productivity ranges.

Twenty-two plant species were selected for the experiment, including four annual species and eighteen perennial species (Table 2). Perennial species were chosen to be representative of the species naturally occurring across the range of productivity within this grassland. Annuals are uncommon at the site, yet are present and could respond to competition differently than perennials [62]. Due to limited seed availability for the annual species present at the site, annual species were chosen from the regional pool and according to seed availability. Combined, these 22 species represent approximately 25% of the total vegetative cover and 15% of the total vascular species richness at the site.

**Table 2:** A list of species used within the experiment by growth form and family.

Life history	Family	Species	Frequency
Annual	Brassicaceae	*Capsella bursa-pastoris*	<1
		*Lepidium densiflorum*	<1
	Chenopodiaceae	*Chenopodium album*	6
		*Monolepis nuttalliana*	<1
Perennial	Apiaceae	*Zizia aptera*	2
	Asteraceae	*Artemisia ludiviciana*	57
		*Gaillardia aristata*	7
		*Heterotheca villosa*	<1
		*Solidago missouriensis*	69
		*Symphyotrichum laeve*	40
	Campanulaceae	*Campanula rotundifolia*	52
	Fabaceae	*Hedysarum alpinum*	5
	Lamiaceae	*Monarda fistulosa*	4
	Linaceae	*Linum lewisii*	<1
	Poaceae	*Bouteloua gracilis*	28
		*Bromus inermis*	26
		*Elymus trachycaulus*	85
		*Nasella viridula*	<1
		*Poa pratensis*	95
	Rosaceae	*Geum triflorum*	23
		*Potentilla arguta*	12
	Scrophulariaceae	*Penstemon gracilis*	14

**Note:** Growth forms were based upon observed morphologies under competition and determined following Cornelissen *et al.* (2003). Frequency of occurrence was determined by a 2009 survey of 100 2×2 m plots spread across the field site. Values of <1 denote plants that are known to occur at the field site, but were not observed within the plots.

### Experimental Design

Twenty replicate blocks, each consisting of two 2 m×2 m plots, were established in the summer of 2008. To test for the effect of neighbours on plant growth and survival, one plot of each pair was randomly assigned a neighbours removed treatment and the other a neighbour intact treatment. Neighbour removal was initially accomplished through a combination of mowing and the application of a broad spectrum herbicide (Round-up®, Monsanto), and maintained by applying herbicide to non-target plants using a paint brush and hand weeding as needed. These plots were surrounded by a 0.5 m vegetation free buffer zone at the edge of which roots were severed to a depth of 0.1 m to minimize interactions with vegetation surrounding the plots. This edge was re-cut on an approximately monthly basis. As the removal of vegetation in neighbours removed plots resulted in the removal of much of the litter layer, neighbours intact plots were also raked to remove an equivalent portion of the litter layer. Standing biomass was low at the time of transplanting, minimizing the amount of damage to aboveground plant structures; however, we kept raking intensity low to reduce damage to the soil surface and existing vegetation.

Each plot was divided into sixty-four 0.25 m by 0.25 m cells in an 8×8 grid. In total there were 20 blocks ×2 plots ×64 cells for a total of 2560 planting locations in this experiment. One seedling of a given species was transplanted into each cell such that each species had two to three individuals in each plot. Species’ positions within the grid were assigned randomly for each block, so that the identity of the planted neighbour was consistent between plots within the block, but varied among blocks. This design ensured that, if competition occurred between seedlings that we planted, any neighbour-specific competitive effects [63], [64] on target plant performance would remain consistent within blocks, but that species-specific responses would not be confounded by the identity of the planted neighbour when comparing across species and across blocks.

Seedlings were started in the greenhouse and transplanted into the field at the beginning of June 2009 at approximately four weeks of age. All seedlings at the time of transplanting had at least their first two true leaves, although most had at least four leaves. When transplanting, a 2 cm wide and 5 cm deep circular hole was made using a soil step sampler and the seedling was inserted along with the propagation soil. The narrow hole diameter was chosen to minimize damage to surrounding roots, although some trampling of the surrounding vegetation did occur. To increase establishment success, plots were watered with approximately 2 L/m^2^/day for the first 5 days following seedling transplanting and 1 L/m^2^/day for the next 5 days, but received no supplemental water after 10 days. All seedlings that died within the first ten days of the initial transplanting were replaced with new individuals from the trays grown in the greenhouse. Seedling mortality was monitored for all plants approximately biweekly following the initial replacement period for transplants, with percent survival of transplants calculated in late August 2009 after 13 weeks of growth.

### Plant Growth

To measure biomass and estimate growth of annual plants, all annuals were clipped in early August 2009 to avoid mass loss due to seed dispersal. Plants were then dried at 65°C for at least 72 h and weighed. This study is intended to persist long-term, and thus destructive measures of perennial plants could not be taken. Instead, perennial growth was estimated using species-specific biomass regressions and plant measurements taken in late August 2009, prior to senescence. For these plants, we measured the maximum width (w_1_), width perpendicular to maximum (w_2_), and height (h) of each plant. We took the same measurements on a second smaller set of plants also grown with and without neighbours, and clipped these plants for the development of our biomass regressions. For 3 of the 18 species, survival of these plants was too low (N<5) to estimate biomass and these species were removed from all analyses concerning plant growth. We used backwards step-wise regression to estimate biomass with ln (biomass) as the dependent variable and ln (basal area), ln (height), and ln (flowering stems) as independent variables. For *Bouteloua gracilis,* our step-wise regression model selected only ln(flowering stems), causing plants without flowers to be underestimated; therefore we removed flowering stems from our starting model, which still gave acceptable results (R^2^ = 0.919). For *Hedyserum alpinum*, we were unable to obtain a suitable regression (P>0.05); therefore we removed *H. alpinum* from analyses related to growth. The full set of equations and the regression results can be found in Table 3. Similar model selection analyses using mixed models and small sample AIC (AIC*_c_*) selected identical parameters and gave identical coefficient estimates as the regression approach. We chose not to harvest roots or monitor root growth due to our desire to avoid destructive sampling and the inherent logistical issues of having more accurate measures of root biomass in the neighbours removed than the neighbours intact treatment [65]. However, our estimates of shoot growth should provide adequate estimates of the total effect of neighbours on plant performance, which presumably is a proxy for fitness [65].

**Table 3:** Biomass regression coefficient estimates and significance tests.

	Regression coefficients				Regression results			
Species	Intercept	ln(height)	ln(flowers)	ln(basal area)	Adjusted R^2^	F	df	P
*Zizia aptera*	−6.76			1.11	0.964	240.61	1,8	<0.001
*Artemesia ludiviciana*	−8.03			1.33	0.944	153.23	1,8	<0.001
*Gaillardia aristata*	−8.92			1.49	0.953	182.42	1,8	<0.001
*Solidago missouriensis*	−11.01	3.85			0.645	15.54	1,7	0.006
*Symphyotrichum laeve*	−7.65		0.47	1.14	0.977	193.27	2,7	<0.001
*Campanula rotundifolia*	−11.01		−1.48	2.10	0.892	29.964	2,5	0.002
*Monarda fistulosa*	−9.14	1.14		0.95	0.991	343.13	2,4	<0.001
*Bouteloua gracilis*	−6.981			1.00	0.903	56.99	1,5	0.001
*Bromus inermis*	−7.01	1.1	0.48	0.54	0.993	437.36	3,6	<0.001
*Elymus trachycaulus*	−11.53	3.32			0.986	572.31	1,7	<0.001
*Nasella viridula*	−8.95	1.86		0.56	0.97	147.94	2,7	<0.001
*Poa pratensis*	−8.21	1.44		0.68	0.966	39.21	2,6	<0.001
*Geum triflorum*	−8.23			1.43	0.979	416.72	1,8	<0.001
*Potentilla arguta*	−7.22	0.62	2.41	0.76	0.971	67.17	3,3	0.003
*Penstemon gracilis*	−7.17		2.39	1.08	0.995	527.78	2,3	<0.001

**Note:** For each species, if a particular regression coefficient was removed from the regression model by backward step-wise regression, then it is left blank in the table below. For *Bouteloua gracilis*, ln(flowers) was not included in the regression model as it caused underestimation of biomass for plants without flowers.

### Productivity Estimation

Aboveground net primary productivity was estimated as standing biomass in grams dry weight/m^2^ (g/m^2^) for each block at peak biomass in late July 2010. We could not harvest biomass from within the plots with neighbours intact as it would disrupt the long term goals of the study. Therefore, vegetation was clipped in four 0.1 m by 1 m quadrats surrounding the block, with individual quadrats placed on the north, south, east and west sides of each block. Samples were then sorted to remove dead material, dried at 65°C for at least 72 h, and weighed. Values for the individual quadrats ranged between 130 and 630 g/m^2^; however, productivity is naturally spatially heterogeneous at the site. We therefore used the average biomass of the four quadrats as our estimate of productivity for that block. Averaging among the quadrats restricted the range of productivity to 225−460 g/m2. This could underestimate the absolute range of productivity between blocks, but should still represent the relative differences among blocks. We recognize that given the range of productivity covered, our test is not a definitive test of the associated theories; however, it is a test of the relationship between competition and productivity for this site.

Soil moisture was measured in both neighbours intact and neighbours removed plots using a ML2x – ThetaProbe Soil Moisture Sensor (Delta-T Devices) in late May 2010. Within each plot soil moisture was measured 5 times, once in each corner and the center. Within neighbours removed plots, care was taken to avoid sampling within the immediate vicinity of a seedling. Soil moisture in neighbours removed plots approximates the moisture retention capacity of the soil (gross water supply), whereas soil moisture where neighbours are intact approximates difference between supply and demand (net water supply) [5]. We did not calculate the ratio of supply to demand as suggested by Taylor [43] because we did not directly measure demand. Similarly, we chose not to estimate demand using the method laid out by Davis [5] due to potentially confounding instances when neighbours increase water availability as seen in studies of facilitation [37]. We recognize that the effect of the plant community on soil moisture in late May is less than can be expected in mid-July, but soil moisture measurements in July were not taken. However, among other plots at the field site within the same growing season, net water supply in May was highly correlated with net water supply in July (r = 0.782, *P*<0.001).

Although neighbour biomass can be expected to vary with water availability, this correlation is not perfect. Across a survey of 100 sites, net water supply was correlated with standing crop (r = −0.321, P = 0.003; J.A. Bennett, unpublished data). However, this does not explain the majority of the variation in standing crop. In this particular study, standing crop was uncorrelated with either net (r = 0.146, *P = *0.538) or gross water supply (r = 0.178, *P = *0.453), although net and gross water supply were highly correlated (r = 0.697, *P = *0.001). This suggests that other factors are potentially limiting to plant growth, including, but not limited to, nitrogen [57]. Determining these factors and their role in competition is the subject of future research.

### Competition Metrics

Competition can differentially affect survival and growth [1], [45], [47]. Further, both survival and growth are important components of fitness [1], [45], [66], [67], with species-specific competitive effects on seedling growth being a strong predictor of species’ abundances in the field [45]. Thus we estimated the effect of competition for both survival and size separately.

The choice of response metric can influence the form of the C–P relationship [1], [30], [50], [68], [69]. We chose the log response ratio (lnRR) to estimate competitive intensity [1], [24] as it is an unbiased estimate of the effect of competition which is usually normally distributed and symmetrical around zero [68], [69]. The ratio was calculated such that positive response ratios indicate competition and negative ratios indicate facilitation:
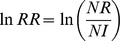



From the response ratios, we classified interactions as positive, neutral or negative to determine interaction frequency. Interactions were classified as positive if neighbours increased plant survival or size by greater than 10% (lnRR<−0.0953) and negative if neighbours reduced survival or size by greater than 10% (lnRR>0.0953). All other interactions were classified as neutral.

We calculated the relative importance of competition using the importance index (*I_imp_*) [70], modified so that competition would be positive and facilitation negative:
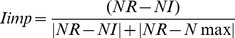



This index was chosen as it is symmetrical around zero for competitive and facilitative interactions although there have been some concerns made regarding its utility in some situations [69]. Here *N_max_* refers to the maximum performance of a given species in either the neighbours removed or neighbours intact treatments. These indices were calculated for each species within each block except under specific conditions. For survival, indices were not calculated for a given species within a specific block if mortality was complete for that species within that block. Similarly, indices were not calculated for size if mortality for a given species reached 100% in either neighbours intact or neighbours removed plots within that block.

### Statistical Analyses

To determine if the effects of competition on survival and size differed, we used two mixed models, one for competitive intensity and one for competitive importance. These models included plant response measure (survival vs. size) as a fixed factor and species and block as random factors, with either competitive intensity or importance as the response variable in SPSS (v18.0). All plant response measures for a given species were pooled at the plot level prior to analysis; survival represents the proportion of seedlings that survived the year and size represents the average size of surviving individuals within that plot.

To test whether neighbour standing crop, gross water supply, or net water supply were associated with changes in competitive intensity or importance for either plant survival or growth, we used twelve mixed models specifying different independent and response variables in SPSS (v18.0). For each independent variable (standing crop, gross water supply, net water supply) we ran four models: lnRR – survival, *I_imp_* – survival, lnRR – growth, and *I_imp_* – growth. Although gross water supply is not technically a measure of productivity, it represents potential productivity. Thus for ease of comparison, we will refer to it as a measure of productivity. Variation in competition-productivity (C–P) slopes among species was accounted for with a random interaction between species and productivity that allowed the C–P slopes to vary randomly by species. Estimation method (regression or weighed) was also included as a random effect for these analyses.

To test for changes in the frequency of interaction types (competitive, neutral or facilitative) across the range of biomass and resource availability found for both plant size and survival, we used six generalized linear mixed models with PROC GLIMMIX in SAS (v9.2) specifying the multinomial distribution. Interaction type was used as the response variable with either neighbour biomass, gross water supply, or net water supply as continuous fixed effects and species as a random effect.

## Results

**Figure 1:**
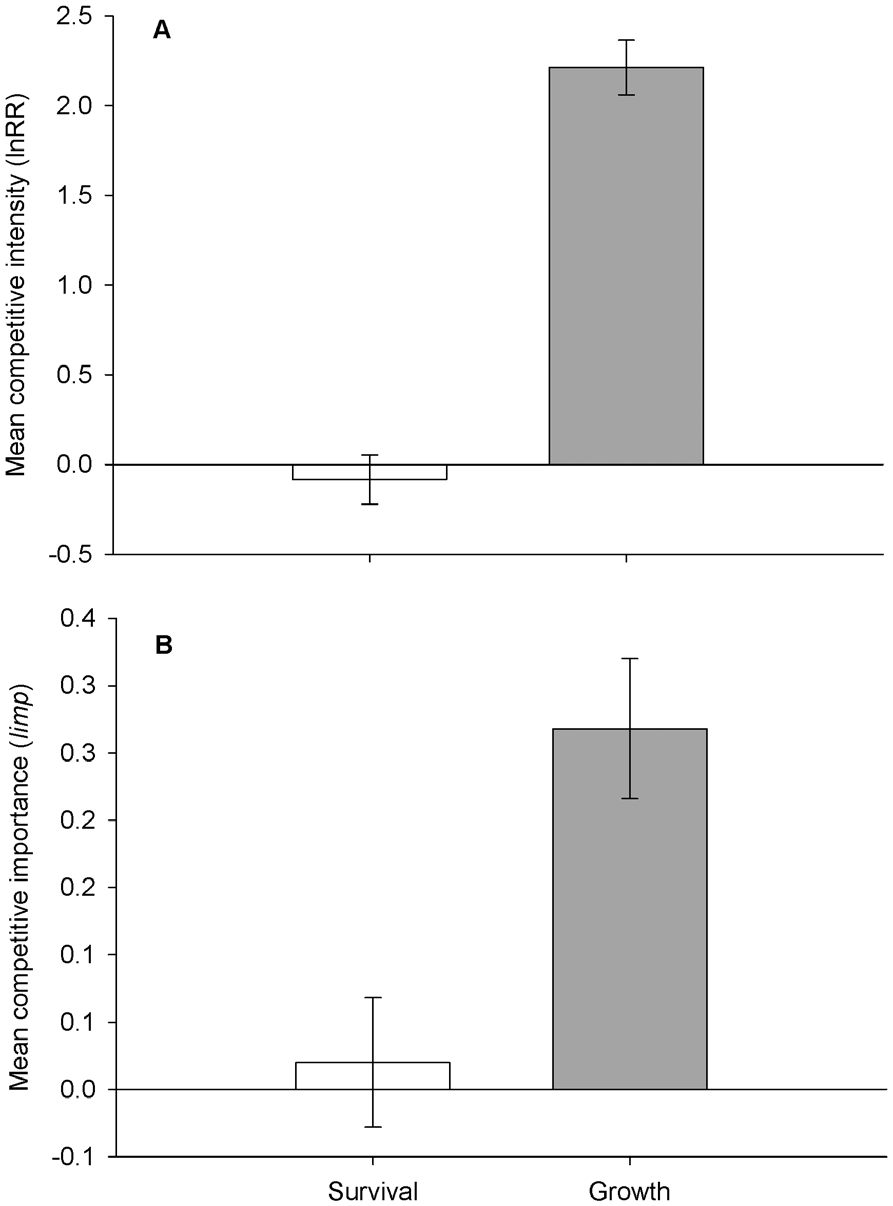
Competitive effects on separate seedling life history aspects. Shown are the mean competitive intensity (A) and competitive importance (B) for seedling survival and size. Responses were calculated such that competition is represented by positive values and facilitation by negative values. Means represent the average of all species and error bars indicate one standard error. Note that the y-axes in the two panels use different scales.

Across all species and blocks, plants were 17.3 times larger in no competition plots than competition plots; whereas survival was 1.2 times higher with neighbours (55% survival) than without (47% survival). This resulted in variation in the magnitude of competitive intensity (F_1,614_ = 420.85, *P*<0.001, Fig. 1A) and importance (F_1,610_ = 49.92, *P*<0.001, Fig. 1B) between survival and size. The magnitude of competitive intensity were comparable to other competition studies on seedlings conducted at the site for both size and survival [57], [58], suggesting these results are ‘typical’ for this location.

**Figure 2:**
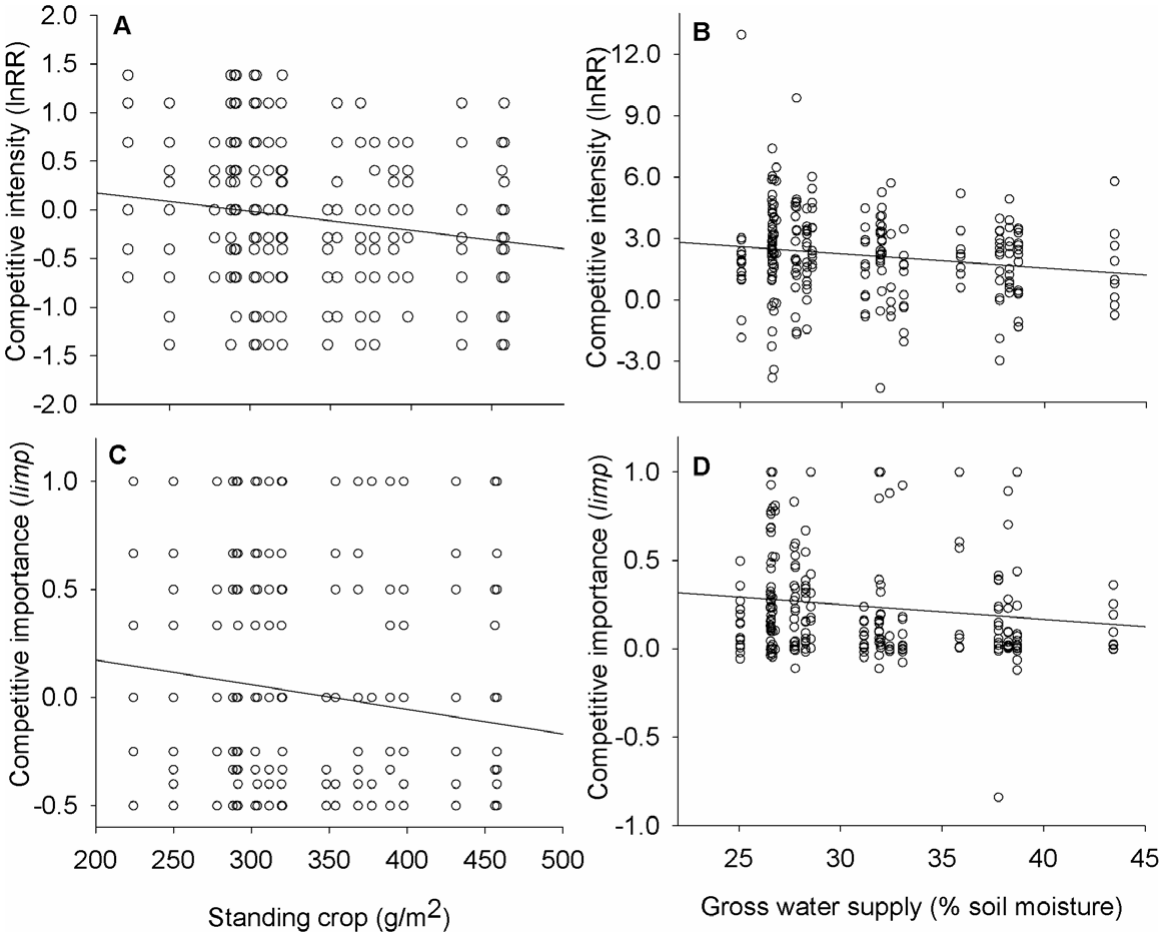
Changes in competitive intensity and importance with productivity. Competitive intensity (A) and relative competitive importance (C) decline as a function of standing crop for survival. Similarly, competitive intensity (B) and importance (D) decline for plant growth with gross water supply. Horizontal solid lines denote zero on the y-axis. Values above this line are competitive and below this line are facilitative. Dashed lines represent best fit lines. Each panel has a different scale for the y axis and that x axes are the same for A and C and for B and D.

Both competitive intensity and importance declined with increasing neighbour biomass when considering seedling survival (Fig. 2A, C, Table 4); however neither gross nor net water supply significantly affected competitive effects on seedling survival (Table 4). For seedling growth, both competitive intensity and importance declined with increasing gross water supply (Fig. 2B, D, Table 4), but neither neighbour biomass nor net water supply had a significant effect (Table 4).

**Table 4:** Results of mixed model analysis of competition productivity relationships.

Plant response	Competition metric	Response variable	F	d.f.	P
Survival	Intensity (lnRR)	Gross water supply	0.69	1,306	0.408
		Net water supply	0.65	1,362	0.422
		**Standing crop**	**18.00**	**1,250**	**<0.001**
	Importance (Iimp)	Gross water supply	0.61	1,359	0.434
		Net water supply	0.12	1,392	0.728
		**Standing crop**	**12.71**	**1,384**	**<0.001**
	Interaction frequency	Gross water supply	1.07	1,381	0.301
		Net water supply	0.49	1,381	0.486
		**Standing crop**	**16.92**	**1,381**	**<0.001**
Biomass	Intensity (lnRR)	**Gross water supply**	**6.55**	**1,225**	**0.011**
		Net water supply	0.15	1,226	0.700
		Standing crop	0.42	1,221	0.516
	Importance (Iimp)	**Gross water supply**	**4.35**	**1,223**	**0.038**
		Net water supply	0.19	1,222	0.666
		Standing crop	0.02	1,225	0.880
	Interaction frequency	Gross water supply	0.32	1,205	0.573
		Net water supply	0.39	1,205	0.536
		Standing crop	0.91	1,205	0.342

**Note:** Significant results are in bold.

Across all species, facilitative interactions were common for plant survival (45%), but rare for size (14%). The remaining neighbour effects on survival were both competitive (30%) and neutral (25%), whereas, neighbours were largely competitive when measuring growth (85%), and rarely neutral (<1%). Echoing the declines in competitive intensity and importance, the frequency of competitive effects on survival decreased with standing crop, with a concurrent increase in the frequency of facilitative effects (Table 4). Interaction frequency was not found to change for survival with either gross or net water supply. Given the rarity of non-competitive effects on plant growth, it is unsurprising that the relative frequency of competitive and facilitative interactions on growth did not change regardless of the productivity estimate used (Table 4).

## Discussion

### Neighbour Interactions and Productivity

Across all models, we found that competitive intensity declined with productivity, but that the type of productivity measurement that was associated with this decline depended on the plant response. However, we found no evidence of increasing competitive effects across the range of productivity used as predicted by Grime [11], [12] and parts of the SGH [37], [39], [40], [51]. This was consistent regardless of the competition metric, plant response, or productivity measurement used. As competition was neither intense nor relatively important at high productivity for either plant survival or size, we suggest that these results are also not consistent with an increase in the likelihood of competitive exclusion as predicted by Newman [13], at least not over this range of productivity.

Given that we found a decline in competitive intensity with at least one measure of productivity for both plant growth and survival, we find little support for Tilman [3], [14] either. However, we did find that competition was invariant when measuring plant growth and standing crop as well as survival and gross water supply. This could be construed as support for Tilman’s prediction, but given that C–P relationships were so often negative, the support is marginal at best.

As previously mentioned, we found little support for the most common prediction of the SGH: that facilitation will increase with increasing stress [36], [37], [39], [40], [42], nor did we see evidence for a hump-shaped distribution of facilitation along a stress gradient [40], [42]. It is possible that a hump-shaped relationship would appear if our range of productivity covered lower productivity areas. However, across the range of productivity measured, facilitation of survival always decreased with stress and we found no evidence of net facilitation in relation to plant growth at any productivity level. Our results are consistent with the earliest version of the SGH [37] which predicted that facilitation could increase with productivity under high herbivore pressure due to associational defences which lead to a reduced risk of herbivory [37], [39]. Although this mechanism is likely active at the site to some extent, it is unlikely to be dominant as herbivory remains low when cattle grazing is not active [61]. However, other mechanisms of facilitation including protection from light damage and desiccation [38] could have led to this pattern (see below).

Although the SGH cannot explain the decline in competitive effects on plant growth and survival with productivity found in this system, these results are consistent with separate meta-analyses [1], [33]. Both our results and the meta-analyses are at least partially consistent with theories that account for variation in both resource supply and demand [5], [43], [71]. The decline in competition with productivity we observed is consistent with his predictions, but we did not find a relationship between competition and net resource supply as predicted by Davis [5]. This suggests that the supply and demand theory is also unable to predict the outcome of competition in this grassland community. However, we cannot rule out this theory because our measurements of net resource supply were not perfectly timed. Although net resource supply is correlated between early and mid-growing season, this correlation is not perfect and it is possible that a relationship exists between net resource supply and competition at peak biomass.

There are other pieces of evidence that suggest that resource supply and demand are important in determining the relative effects of competition. Nutrient uptake is not perfectly efficient [72] and light is not always limiting at high productivity [73], [74]. This can result in increasing net nutrient availability at high productivity [72], which does not necessarily coincide with an increase in aboveground competition [73], [74], and can cause a decrease in total competition with productivity. Of course an increase in aboveground competition is going to be dependent on the range of productivity explored. However, even if at higher productivity, competition does increase, this would suggest that the relationship between competition and productivity would have to be non-linear for this site.

We also found a number of differences between plant survival and growth which are consistent with previous findings; neighbours tend to have neutral to positive effects on plant survival and competitive effects on plant growth [40], [45], [46], [75]. We also found that neighbour effects were associated with resource supply for growth and standing crop for survival. These differences between seedling survival and growth are consistent with the concept that environmental effects on plant-plant interactions can vary depending on the life history stage of the plant [40], [47], [48], [49]; however, this aspect of theory is not fully developed.

Some have hypothesized that neighbour effects can become more competitive as plants grow, in part due to differences in resource requirements [48]. In the current study, the association between resource supply and competitive effects on growth suggests that neighbour effects on growth are largely determined by resources. Resource interactions in mild environments are thought to be mostly negative [40], which explains the large competitive effects on growth. However, seedling survival can be facilitated through a number of mechanisms including reduced probabilities of desiccation, photoinhibition and herbivory [38]. These mechanisms do not necessarily affect available resources, which may explain why seedling survival increased with standing crop and not resource supply. In some cases, this can lead to an increase in facilitation of seedling survival with productivity as seen in this study and others [1], perhaps because low productivity sites have too little vegetative cover to provide these benefits to seedlings, making facilitation more likely at higher productivities.

Neither our results nor those of the meta-analyses [1], [33] fully support any of the major theories, suggesting that new theory regarding the relationship between plant-plant interactions and environmental gradients should be developed. These theories should incorporate both competition and facilitation as both are occurring simultaneously within most sites and should also account for the effects of multiple environmental gradients on different life history components. Some work has been done in this direction [2], [40], although these theories must become more mechanistic and explicit in their predictions.
